# Engineering *Saccharomyces pastorianus* for the co-utilisation of xylose and cellulose from biomass

**DOI:** 10.1186/s12934-015-0242-4

**Published:** 2015-04-28

**Authors:** William Kricka, Tharappel C James, James Fitzpatrick, Ursula Bond

**Affiliations:** Moyne Institute, School of Genetics and Microbiology, Trinity College Dublin, College Green, Dublin 2, Ireland

**Keywords:** *S. pastorianus*, Co-utilisation of xylose and cellulose, Biomass, Spent grain fermentations

## Abstract

**Background:**

Lignocellulosic biomass is a viable source of renewable energy for bioethanol production. For the efficient conversion of biomass into bioethanol, it is essential that sugars from both the cellulose and hemicellulose fractions of lignocellulose be utilised.

**Results:**

We describe the development of a recombinant yeast system for the fermentation of cellulose and xylose, the most abundant pentose sugar in the hemicellulose fraction of biomass. The brewer’s yeast *Saccharomyces pastorianus* was chosen as a host as significantly higher recombinant enzyme activities are achieved, when compared to the more commonly used *S. cerevisiae*. When expressed in *S. pastorianus,* the *Trichoderma reesei* xylose oxidoreductase pathway was more efficient at alcohol production from xylose than the xylose isomerase pathway. The alcohol yield was influenced by the concentration of xylose in the medium and was significantly improved by the additional expression of a gene encoding for xylulose kinase. The xylose reductase, xylitol dehydrogenase and xylulose kinase genes were co-expressed with genes encoding for the three classes of *T. reesei* cellulases, namely endoglucanase (EG2), cellobiohydrolysase (CBH2) and β-glucosidase (BGL1). The initial metabolism of xylose by the engineered strains facilitated production of cellulases at fermentation temperatures. The sequential metabolism of xylose and cellulose generated an alcohol yield of 82% from the available sugars. Several different types of biomass, such as the energy crop *Miscanthus sinensis* and the industrial waste, brewer’s spent grains, were examined as biomass sources for fermentation using the developed yeast strains. Xylose metabolism and cell growth were inhibited in fermentations carried out with acid-treated spent grain liquor, resulting in a 30% reduction in alcohol yield compared to fermentations carried out with mixed sugar substrates.

**Conclusions:**

Reconstitution of complete enzymatic pathways for cellulose hydrolysis and xylose utilisation in *S. pastorianus* facilitates the co-fermentation of cellulose and xylose without the need for added exogenous cellulases and provides a basis for the development of a consolidated process for co-utilisation of hemicellulose and cellulose sugars.

**Electronic supplementary material:**

The online version of this article (doi:10.1186/s12934-015-0242-4) contains supplementary material, which is available to authorized users.

## Background

Decreasing crude oil reserves and increasing costs of extracting the limited supply of shale gas production through hydraulic fracturing have given increased momentum to finding alternative cost-effective forms of renewable energy sources. Bioethanol and biodiesel can be produced from renewable biomaterials and a variety of food crops including sugarcane, corn and soya bean have been used. However the large-scale use of food crops for fuel may cause long-term food scarcity. An alternative approach for producing biofuels is the use of carbohydrates extracted from non-food biomass [1,2]. Plant biomass is mainly composed of lignocellulose, which contains three major components cellulose, hemicellulose and lignin. The relative amount of each component varies in different plant types, with an average composition of cellulose 30-50%, hemicellulose 20-30% and lignin 15-25% [3].

Cellulose, a β-glucan linear polymer of D-glucose linked by β-1,4-glycosidic bonds, is the most abundant polysaccharide on earth [4]. In plants, it is organised into crystalline fibers containing tightly packed microfibrils of approximately 30 β-glucan chains containing some disorganised amorphous regions [5]. Hemicellulose is a highly branched heteropolymer composed of pentose and hexose sugars such as xylose, arabinose, mannose, glucose and galactose as well as certain sugar acids [6]. The composition of hemicellulose is variable and depends upon the plant source however glucose and xylose are generally the major hexose and pentose sugars respectively [7]. The third component of lignocellulose, lignin, is a polymer of phenylpropanoid monomers. Lignin links both hemicellulose and cellulose together forming a physical barrier in the plant cell wall [8]. Unlike cellulose and hemicellulose, hydrolysis of lignin does not generate fermentable sugars.

Fermentable sugars, mainly glucose and xylose, can be readily extracted from the hemicellulose fraction of biomass through physical and/or chemical pre-treatments [9]. Glucose is readily metabolised by most organisms via glycolytic pathways. Xylose metabolism occurs mainly in fungal and bacteria species via a pathway requiring two oxidoreductases, xylose reductase (XR) and xylitol dehydrogenase (XDH) or alternatively through the action of a single enzyme, xylose isomerase (XI) [10-13]. The product of both pathways, xylulose, is phosphorylated by xylulose kinase (XKS) and then enters the Pentose Phosphate Pathway (PPP), generating intermediates of the glycolytic pathway [2].

Due to its crystalline nature, the cellulose fraction of biomass is more recalcitrant to sugar extraction, therefore, additional enzymatic hydrolysis, involving the concerted action of three major classes of cellulases, namely endoglucanases (EG), exoglucanases or cellobiohydrolases (CBH) and β-glucosidases (BGL) is required [2,14]. Endoglucanases cleave the cellulose backbone randomly at amorphous sites along the cellulose fibre exposing new chain ends. Cellobiohydrolases act processively on reducing and non-reducing chain ends to release mainly the disaccharide cellobiose. Finally, β-glucosidases hydrolyse the β-1,4 glycosidic bond of cellobiose and cellooligosaccharides to release glucose.

The microorganisim of choice for industrial bioethanol production is the ethanologenic Baker’s yeast *Saccharomyces cerevisiae,* due to its long fermentation history, high ethanol yields and general robustness to environmental stresses encountered during industrial fermentations. However, *Saccharomyces* species cannot metabolise either xylose or cellulose. While a few natural *Saccharomyces* isolates have been identified that grow slowly in medium containing xylose as a sole carbohydrate source and several genes encoding putative xylose-utilising enzymes have been identified in the genomes of *Saccharomyces* species [15,16], xylose metabolism by *Saccharomyces* species is inefficient and unsuitable for industrial processes and most likely serves as a fail-safe mechanism for survival under conditions when nutrients are limiting. To date genes encoding cellulases have not been identified in yeasts.

To overcome these problems, a recombinant strategy has been pursued in which genes encoding for xylose and cellulose metabolism have been introduced into *Saccharomyces* species (reviewed in [2,7]). Relatively efficient xylose metabolism has been achieved in yeasts by expressing xenotropic XR/XDH or XI genes combined with overexpression of the host encoded XKS gene [17-25]. Further improvements in yields have been obtained through adaptive or directed evolutionary strategies aimed at increasing metabolic fluxes through the PPP or altering co-factor requirements for the xylose utilising enzymes [17,26-30]. Likewise, recombinant cellulases, in secreted- or cell tethered-forms, have been produced in yeasts to allow for extracellular hydrolysis of cellulose [2,31-39]. Fermentations using cellulose as a sole carbohydrate source are hampered by a ‘chicken and egg’ conundrum, as cellulase production and secretion by the recombinant yeast requires cell growth but this is limited at early stages of fermentation by the lack of fermentable sugars [2]. This problem is currently overcome by the addition of exogenous cellulases to the fermentation medium, however this adds substantial costs to the process.

While *S. cerevisiae* remains the host of choice for heterologous expression of cellulases and xylose utilising enzymes, we recently demonstrated that up to ten times higher levels of cellulase activity is attained from recombinant proteins expressed in the brewer’s yeast *Saccharomyces pastorianus*, when compared to the levels produced in *S. cerevisiae* strains [35].

To date, alcohol production from either cellulose or xylose has been examined separately using *S. cerevisiae* engineered to produce either cellulases or xylose utilising enzymes respectively, however for efficient and economic conversion of biomass to bioethanol, the utilisation of both the hemicellulose and cellulose fractions of lignocellulose will be required. As a first step to generating yeast strains with a capacity to ferment hemicellulosic and cellulosic sugars, genes encoding β-glucosidase (*bgl1*) together with the xylose utilising genes *xr/xdh/XKS* have been introduced into *S. cerevisiae* [40,41]. Alcohol production from xylose and cellobiose using the engineered strains was observed however for fermentation of cellulose, the engineered system still required the addition of exogenous cellulases [42].

To generate a yeast strain for fermenting both xylose and cellulose from biomass, without the need for added exogenous cellulases, we reconstituted the complete enzymatic pathways for cellulose degradation and xylose utilisation in a stress-tolerant strain of *S. pastorianus* by co-expressing all three classes of cellulases genes (*bgl1, eg2, cbh2*) with the *xyl1/xdh1/XKS* genes*.* In fermentations using xylose and cellulose as sole carbohydrate sources, the engineered strains initially utilises xylose, facilitating cell growth and the synthesis and secretion of cellulases, allowing for continued fermentation and alcohol production from cellulose, without the need for added exogenous cellulases.

## Results

### Choice of host

**Figure 1:**
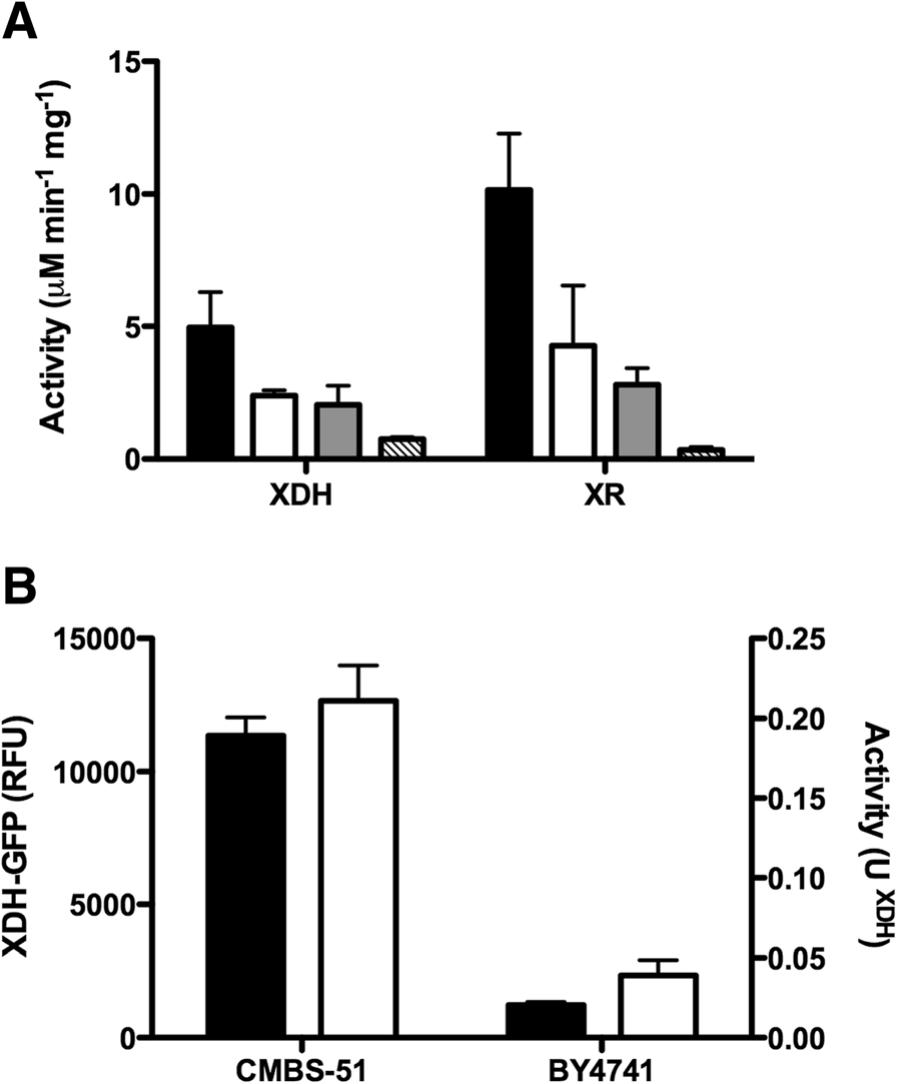
Production of xylose utilising enzymes in different yeast species and strains. **A**. Enzyme activities of XR and XDH in *S. pastorianus* (CMBS-51), ; *S. eubayanus*, ; *S. cerevisiae* (L6440;4 N), ; *S. cerevisiae* (BY4740;1 N), . Activities are expressed as NADPH (U^XR^) or NADH (U^XDH^) μM min^−1^ mg^−1^ cell extract. The mean from three independent experiments are shown and error bars show the standard deviation from the mean. **B**. Relative fluorescent units,  (left Y-axis) and XDH activity (μM min^−1^ mg^−1^),  (right Y-axis) in CMBS-51 and BY4741 expressing XDH-GFP. The mean from two independent experiments are shown, error bars shown the standard deviation from the mean.

We previously observed that expression of recombinant cellulase genes in the brewer’s yeasts, *S. pastorianus,* produced significantly higher enzyme activities when compared to the activity observed in *S. cerevisiae* [35]. *S. pastorianus* are generally tetraploid hybrid yeasts containing both *S. cerevisiae* and *S. eubayanus* genomes [43-45]. To determine if a similar enhancement of enzyme activity could be achieved for the xylose utilising enzymes, we compared the activity of *T. reesei xyl1* and *xdh1* genes in haploid and tetraploid isogenic *S. cerevisiae* strains, and in the tetraploid *S. pastorianus,* CMBS-51. The latter is a stress-tolerant derivative of a typical lager yeast CMBS-33. The activities of both enzymes were significantly higher in *S. pastorianus* when compared to the activities in the haploid or tetraploid *S. cerevisiae* strains (Figure 1A). Since it was possible that increased enzyme activity in *S. pastorianus* resulted from the presence of the *S. eubayanus* genome, we also examined the activities in the parental strain *S. eubayanus*, however in this species, the enzyme activities were also substantially lower than that observed in *S. pastorianus* (Figure 1A). Using an *XDH*-GFP fusion protein (Table 1), we observed that increased enzyme activity directly correlated with increased recombinant protein levels (Figure 1B).

**Table 1:** **Gene cassettes**

**Plasmid**	**Primers**		**Starting plasmid**	**Insertion site**
TEFbgl1*	bgl1_Rec1F	bgl1_Rec2R	pRS42H	Sal1
PGKbgl1*	bgl1_Rec1F	bgl1_Rec2R	pRS42H	Sal1
TEFcbh2*	cbh2_Rec1F	cbh2_Rec2R	pRS42H	Sal1
PGKegl2*	egl2_Rec1F	egl2_MidR	pRS42H	Sal1
	egl2_MidF	egl2_Rec2R		
PGK*xyl1*	xyl1F	xyl1R	pRS42H_PGK*bgl1*	Sal1
PGK*xdh1*	xdhF	xdhR	pRS42H_PGK*bgl1*	Sal1
PGK*xi*	xiF, xiMidF	xiMidR, xiR	pRS42H_PGKbgl1	Sal1
PGK*xdh1*GFP	xdhF, GFPF	xdh1GFPR, GFPR	pRS42H_PGKbgl1	Sal1
TEF*XKS1*	Psi_TEFF, xks_MidF	xks_MidR, Psi_CycR	pRSK42K	Psi1
PGK*xyl1*-PGK*xdh1*	Psi_PGKF xr_MidF	xr_MidR Psi_CycR	pRS42H_PGK*xdh1*	Psi1
PGK*xyl1*-TEF*XKS1*-PGK*xdh1*	Acc_TEFF, xks_MidF	xks_MidR, AccR	pRS42H_PGK*xyl1*-PGK*xdh1*	Acc65I
PGK*xyl1*-TEF*bgl1*-PGK*xdh1*	Acc_TEFF, bgl1_MidF	bgl1_MidR, AccR	pRS42H PGK*xyl1*-PGK*xdh1*	Acc65I
PGK*xyl1*-TEF*cbh2*-PGK*xdh1*	Acc_TEFF, cbh2_MidF	cbh2_MidR, AccR	pRS42H PGK*xyl1*-PGK*xdh1*	Acc65I
PGK*xyl1*-PGK*egl2*-PGK*xdh1*	Acc_PGKF, egl2_MidF	egl2_MidR, AccR	pRS42H PGK*xyl1*-PGK*xdh1*	Acc65I

*Reference [35], all other strains generated in this study.

### Comparison of the XR/XDH and XI pathways in *S. pastorianus*

**Figure 2:**
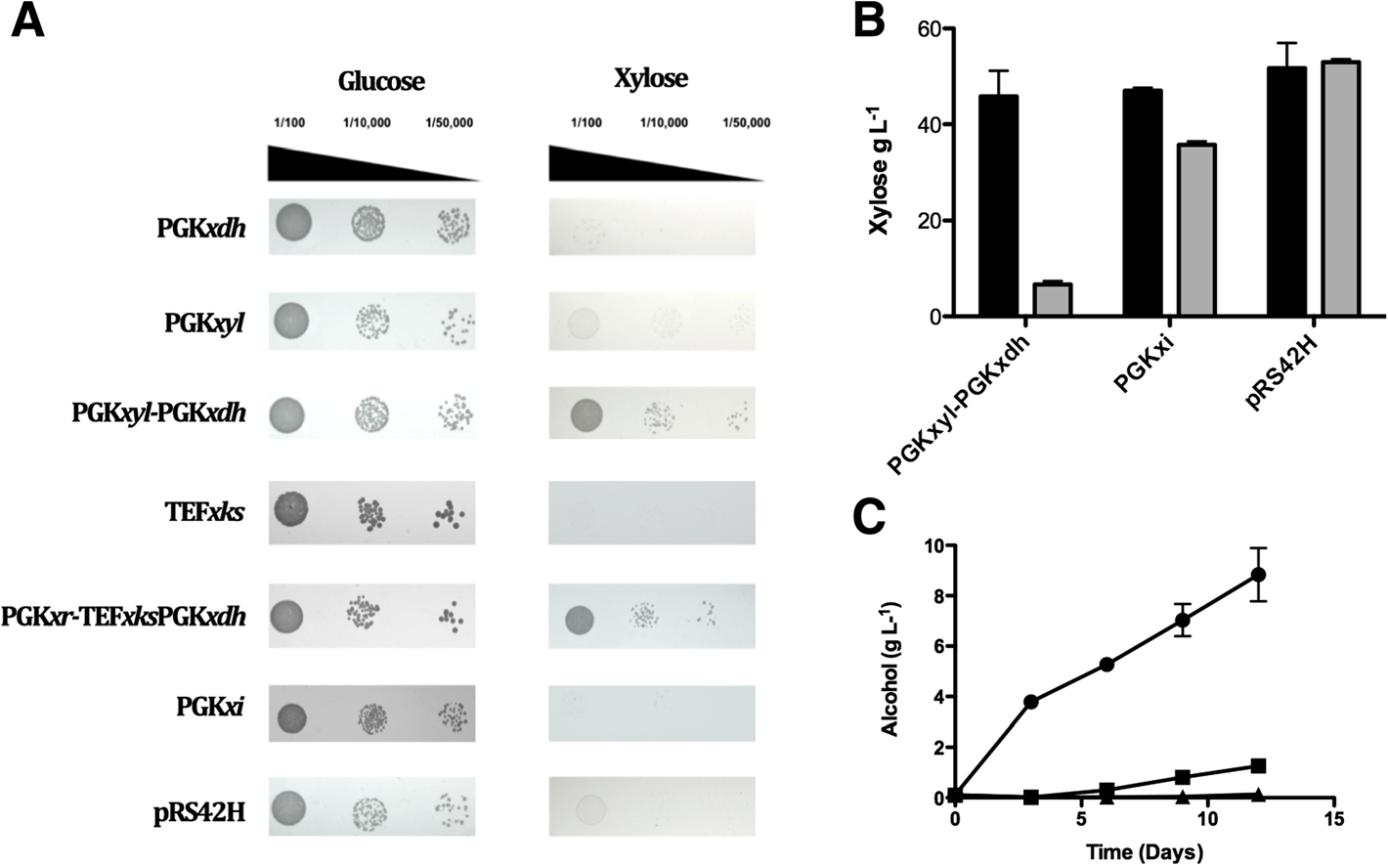
Xylose metabolism in *S. pastorianus*. **A**. *S. pastorianus*, carrying gene cassettes (shown on the left; Table 1) coding for *T.reesei* xylose metabolising enzymes, were grown initially in YEPD. The cells were harvested, washed and resuspened in SC-glucose or SC-xylose media. Various dilutions of the cells were plated onto SC-glucose and SC-xylose agar plates. **B**. Xylose utilization in *S. pastorianus* expressing *xdh1/xyl1*, x*i*, or the empty vector pRS42H. Xylose concentration (g L^−1^) in the medium on Day 0, , and Day 12,  are shown. **C**. Alcohol (g L^−1^) produced by *S. pastorianus* (CMBS-51) expressing *xdh1/xyl1* , *xi*, , or the empty vector pRS42H, . Fermentations were carried out in YEP supplemented with 50 g L^−1^ xylose. The values represent the mean of duplicate independent experiments and error bars show the standard deviation from the mean.

The XR/XDH oxidoreductase pathway requires the co-factors NADPH and NAD^+^ in the forward reactions respectively. This can lead to redox imbalances within the cell resulting in inefficient xylose utilisation. The alternative XI pathway allows xylose metabolism in a single step and has no co-factor requirement [7]. We compared xylose metabolism by the two pathways in *S. pastorianus* expressing the respective genes. Cells co-expressing the *xyl1/xdh1* genes were capable of growing on xylose while expression of either gene individually did not support growth (Figure 2A). Cells expressing *xi* were unable to grow on SC-xylose (Figure 2A), however, xylose utilisation, *albeit* in low levels, was detected when the strain was cultured in rich YEP media with xylose as the sole carbohydrate source (Figure 2B). Xylose utilisation was substantially higher in cells expressing the *xyl1/xdh1* genes (Figure 2B), which produced up to 7 times more alcohol, with levels reaching 8.8 g L^−1^ (Figure 2C).

**Figure 3:**
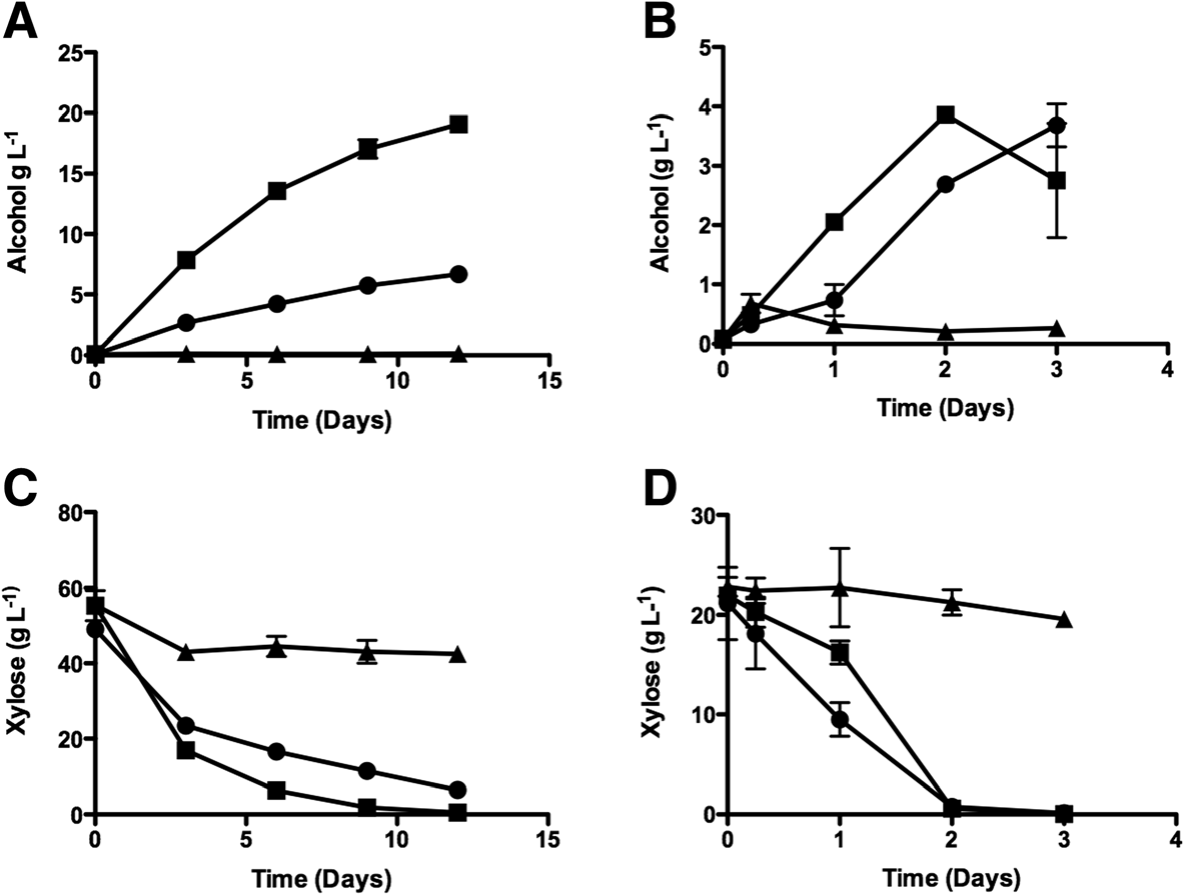
Co-expression of *XKS* improves alcohol yield in *S. pastorianus*. Alcohol(g L^−1^) production **(A, B)** and xylose utilization **(C, D)** by *S. pastorianus* (CMBS-51) expressing *xyl1*/ *xdh1/*XKS (); *xyl1/xdh1* (); or the empty vector pRS42H (). Fermentations in YEP supplemented with 50 g L^−1^ **(A and C)** or 20 g L^−1^ **(B and D)** xylose were carried for 12 days and samples taken at the intervals shown. The values represent the mean of triplicate independent experiments and error bars show the standard error from the mean.

While the XR/XDH pathway produced more alcohol than the XI pathway, the alcohol yield from xylose was still relatively low (39%). To improve this yield, the native *S. cerevisiae* xylulose kinase gene, *XKS1,* was overexpressed in *S. pastorianus* in conjunction with the *xyl1*-*xdh1* genes. The additional expression of *XKS1* increased alcohol levels approximately 3-fold, representing a 64% conversion of xylose to alcohol (Figure 3A). The increased production of alcohol in the presence of *XKS1* was dependent on the starting concentration of xylose. Lowering the starting concentration of xylose from 5% to 2% abrogated the influence of *XKS1* overexpression on the final alcohol yield, however the rate of alcohol production at the lower xylose concentration was still faster with *XKS1* over-expression (Figure 3B). Growth on xylose (Figure 2A) and the rate of xylose uptake from the medium was similar in cells expressing *xyl1/xdh1* and *xyl1/xdh1/XKS1* at both low (2%) and high concentrations (5%), however at the lower concentrations, xylose was more rapidly depleted from the medium (Figure 3C and D).

### Ethanol production from xylose and cellulose fermentations

To optimise for alcohol production from biomass, it is essential that sugars be extracted from both the hemicellulose and cellulose fractions. We previously cloned and expressed copies of the *T. reesei* genes *egl2, bgl1* and *cbh2*, encoding for the three classes of cellulase enzymes, into *S. pastorianus* and demonstrated that the recombinant enzymes act synergistically to degrade the cellulose substrate, PASC [35]. To develop a consolidated process for the utilisation of both hemicellulose and cellulose sugars, each of the cellulase genes were co-expressed with the *xyl1/xdh* genes on a single plasmid (Table 1). The *XKS1* gene was supplied *in trans* on a separate plasmid (Table 1). The activities of XR and XDH were not affected by the additional expression of the cellulase genes on the same plasmid and likewise cellulase activities were unaffected by co-expression of the *xyl1* and *xdh1* (data not shown).

**Figure 4:**
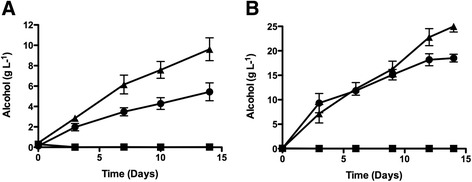
Co-fermentation of xylose and cellulose (PASC). *S. pastorianus* (CMBS-51) expressing the gene cassettes **(A)** PGK*xyl1-*TEF*bgl1-*PGK*xdh1*, PGK*xyl1-*TEF*cbh2-*PGKxdh1 and PGK*xyl1-*PGK*egl2-*PGK*xdh1* or **(B)** additionally PGK*XKS in trans* were individually cultured in YEPD. Cells were harvested, combined in a 1:1:1 ratio and used to inoculate fermentations containing PASC (), xylose (), or PASC and xylose (). The concentrations of PASC and xylose were 412 g L^−1^ and 40 g L^−1^ for **(A)** and 412 g L^−1^ and 50 g L^−1^ for **(B)** respectively. Fermentations were conducted at 30°C for 14 days and samples were taken at the intervals shown and alcohol content (g L^−1^) assayed. The values represent the mean of triplicate **(A)** or quadruplicate **(B)** independent experiments and the error bars represent the standard error from the mean.

Fermentations were carried out using co-cultures of *S. pastorianus* containing the triple expressing plasmids (PGK*xyl1*-TEF*bgl1*-PGK*xdh1*, PGK*xyl1*-TEF*cbh2*-PGK*xdh1* and PGK*xyl1*-PGK*egl2*-PGK*xdh1*; Table 1) in medium containing xylose only, PASC only or both as carbohydrate sources. Yeast cultures grown on PASC as a sole carbohydrate source produced little if any alcohol due to the lack of enzyme activity and cell growth at a fermentation temperature of 30°C [35] (Figure 4A). Cultures grown on xylose produced up to 5.4 g L^−1^ alcohol, however, fermentations containing both xylose and PASC produced almost twice as much alcohol (9.6 g L^−1^), indicating that xylose metabolism supports cellulase production by yeasts, thus enabling a co-fermentation of both xylose and cellulose at mesothermal fermentation temperatures. The additional co-expression of the *XKS1* gene on a separate plasmid (TEF*XKS1*, Table 1) together with the triple expressing plasmids (PGK*xyl1*-TEF*bgl1*-PGKxdh1*,* PGK*xyl1*-TEF*cbh2*-PGK*xdh1* and PGK*xyl1*-PGK*egl2*-PGK*xdh1*) substantially increased alcohol production, increasing the yield 2.6-fold (Figure 4B). In cells overexpressing *XKS1*, xylose is metabolised in preference to cellulose at the early stages of the fermentation. Cellulose metabolism is not observed until day 10 and thereafter alcohol production for the mixed sugars (xylose and PASC) is statistically different from that produced from xylose alone (Figure 4B). The alcohol yield from PASC, xylose, and mixed sugars (xylose and PASC) was 0.14%, 73.5%, and 82.2% respectively at the end of the fermentation. Since the maximum yield of ethanol from PASC at the concentration used is 5 g L^−1^, the increased yield of ethanol in the mixed sugars fermentation is accounted for by complete hydrolysis of the PASC present.

### Sugar extraction from energy crops and industrial waste

Several different types of biomass were examined as sugar sources for fermentation using the developed yeast strains. The energy crops, *M. sinensis*, *M giganteus* and *E. nitens,* as well as industrial waste in the form of spent grains from both industrial and microbreweries were pre-treated as described in the Methods section and the glucose and xylose content of the extracted liquor was determined (Table 2). Increasing the starting concentration of biomass resulted in an increase of the concentration of extracted sugars (data not shown). The xylose content of the liquor was relatively similar in all biomass sources with the exception of *M. giganteus* which showed lower levels in both stem and leaf portions. The glucose content of the different biomass sources was more varied. The highest glucose content was found in spent grains, however there was a high variability between batches of spent grains sourced from different breweries with glucose concentrations ranging from 5.3 – 31 g L^−1^ (Table 2). The *E. nitens* biomass showed the lowest glucose content.

**Table 2:** **Glucose and xylose extraction from biomass**

**Biomass (100 g L)**	**Xylose g L** ^**−1**^ **(sd)**	**Glucose g L** ^**−1**^ **(sd)**
Spent Malt Grain	11.64 (2.13)	16.97 (13.11)
*M.sinensis* Leaf	11.82 (0.65)	3.70 (0.47)
*M.sinensis* Stem	12.18 (1.10)	9.56 (2.55)
*M.giganteus* Leaf	8.33 (1.06)	6.25 (2.24)
*M.giganteus* Stem	4.98 (1.86)	11.19 (0.28)
*E. nitens*	15.71 (0.64)	0.98 (0.09)

sd; standard deviation.

### Alcohol production from spent grains

**Figure 5:**
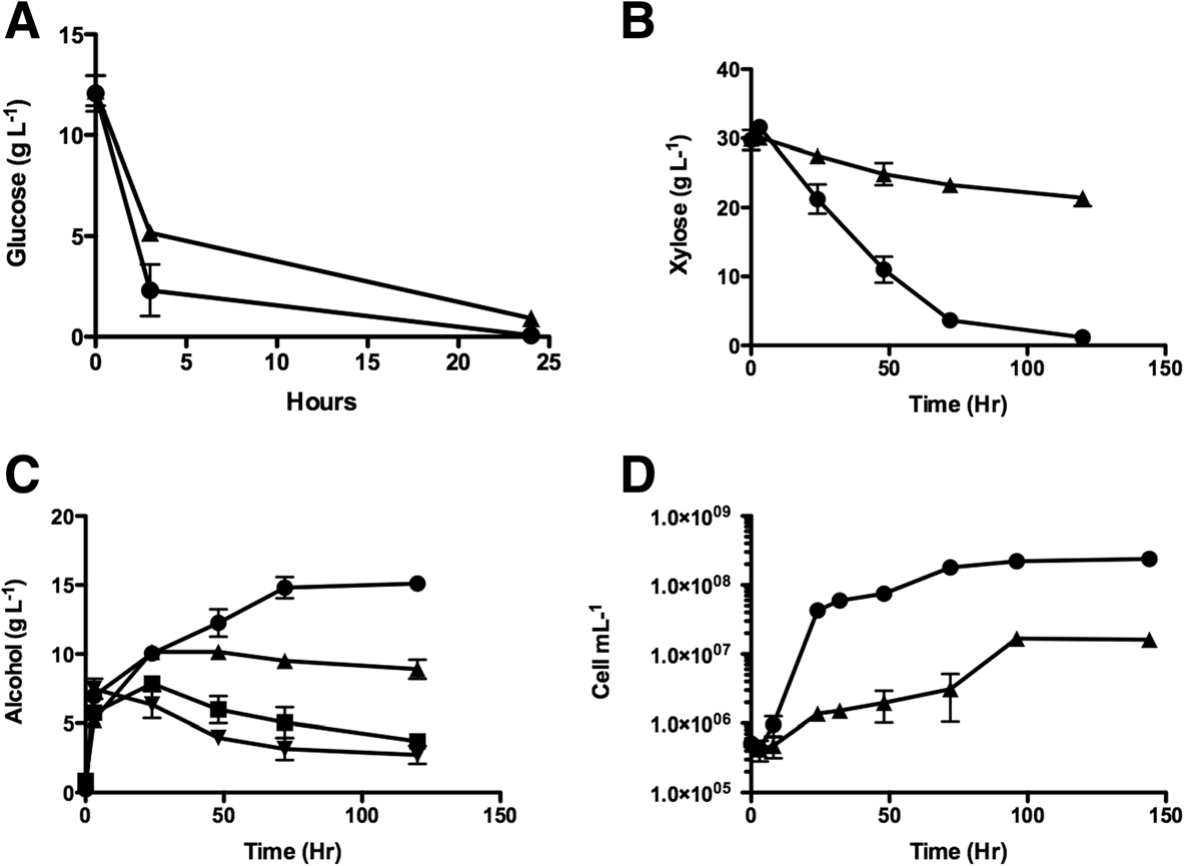
Fermentation of pre-treated biomass liquor. Fermentations were carried out with *S. pastorianus* strains expressing the gene cassettes PGK*xyl1-*TEF*XKS-*PGK*xdh1* in YEP media supplemented with spent grain liquor () or with purified sugars (xylose 30 g L^−1^ and glucose 11 g L^−1^) (). Fermentations were conducted at 30°C for 5 days and samples were taken at the intervals shown and assayed for **A**. Glucose (g L^−1^), **B**. Xylose (g L^−1^) and **C**. Alcohol (g L^−1^). Control fermentations with *S. pastorianus* containing the empty vector pRSH42 in spent grain liquor () or with purified sugars () are also shown in **C**. Panel **D**; cell growth profile of *S. pastorianus* in SGL,  and mixed sugar (xylose and glucose),  at various times. Cells were inoculated at low cell density (1x10^5^ cell/mL). Mean values from triplicate experiments are shown and error bars indicate the standard error from the mean.

Due to its relatively high xylose and glucose content, spent grain liquor (SGL) was chosen for further analysis as a medium for fermentation. For consistency, fermentations were conducted using the liquor from a single batch of spent grain. Glucose and xylose consumption, and alcohol production were compared between fermentations carried out with SGL or with a mixture of purified xylose and glucose at the same concentrations as found in the SGL (Figure 5). Glucose was rapidly consumed within 24 hours from both the purified sugar mixture and the SGL, although a slight lag was observed in the latter fermentation (Figure 5A). Xylose consumption in both fermentations was much slower than glucose consumption: in the purified sugar mixture, xylose was not depleted until 120 hrs (Figure 5B). Strikingly, xylose consumption was severely inhibited in the SGL fermentation (Figure 5B). While up to 15 g L^−1^ alcohol was produced from the xylose/glucose fermentation (Figure 5C), the SGL fermentations produced 10 g L^−1^ alcohol after 24 hours with levels decreasing slightly thereafter, suggesting some degree of alcohol catabolism at late stages of the fermentation (Figure 5C). The alcohol consumption appears to result from a lack of xylose metabolism as fermentations carried out with yeast cells containing the empty vector showed a similar marked alcohol consumption at the later stages of fermentation (Figure 5C), indicating that xylose metabolism allows continued alcohol production in mixed sugar and SGL fermentations.

The lower level of alcohol production in the SGL fermentation appears to be due to cell growth inhibition. This growth retardation is evident when SGL fermentations were inoculated with yeast cultures at low cell densities (Figure 5D). At the high cell densities used in the actual fermentations, very little cell growth is observed, nevertheless, even at these high cell densities, the growth in SGL and mixed sugar fermentations was significantly different (data not shown). The inhibition of cell growth did not affect enzyme production as enzyme activity per cell was not significantly different in mixed sugar and SGL fermentations (data not shown).

## Discussion

For the efficient use of biomass for bioethanol production, it is essential that sugars from both the hemicellulose and cellulose fractions of lignocellulose be utilised. Here, we developed yeasts strains capable of co-utilising cellulose and xylose, the most abundant pentose sugar in the hemicellulose fraction of biomass as carbohydrate substrates.

While *S. cerevisiae* remains the host of choice for heterologous expression of recombinant proteins, we confirm our previous finding that the enzyme activity of recombinant proteins is substantially higher in the lager yeast *S. pastorianus*. Enzyme activity of *xyl1* and *xdh1* were significantly higher in *S. pastorianus* compared to the activities achieved in a haploid *S. cerevisiae* strain or an isogeneic tetraploid strain or in *S. eubayanus*, suggesting that both the ploidy of the strains and the genome content are contributing to the observed increased enzyme activities. Increased enzyme activity appears to result from increased recombinant protein levels in *S. pastorianus* as assessed by XDH-GFP fusion proteins*.* The basis for the increased recombinant protein levels in *S. pastorianus* remains unclear at present but may result from slower protein turnover as a consequence of the slow growth rate of *S. pastorianus* at 30°C [35].

Several problems have been encountered in developing yeast strains capable of ultilising xylose. The redox imbalance resulting from the XR/XDH pathway remains a significant barrier to complete xylose utilisation particularly under strict anaerobic conditions. For this reason, several groups have examined the use of the XI pathway for xylose utilisation. Here we compared xylose utilisation via the two pathways in *S. pastorianus.* Higher alcohol levels are generated from xylose by the XR/XDH pathway compared to the XI pathway confirming previous findings using *S. cerevisiae* as a host [46,47].

We uncovered several factors that influence xylose utilisation in *S. pastorianus*. Overexpression of the *S. cerevisiae XKS1* gene in *S. pastorianus* coupled with increasing the xylose concentration in the medium substantially improved alcohol production. High starting concentrations of xylose appear to allow cells to remain in a fermentative state for longer thus promoting alcohol production. Taken together, the data suggests that efficient xylose utilisation requires both high substrate concentrations and efficient forward reactions to drive metabolic fluxes towards alcohol production.

We used the previously developed *S. pastorianus* strains to examine the co-utilisation of xylose and cellulose. Hydrolysis of cellulose and its use a sole carbohydrate source in mesothermal fermentations is severely impeded by a classic “chicken and egg” conundrum: for cellulase production, cell growth is required but on the other hand, cell growth is retarded by the lack of available fermentable sugars prior to cellulose hydrolysis. Additionally, optimum activity of cellulases is achieved at temperatures in the range of 50-60°C and is significantly reduced at 30°C, the optimum temperature for mesophilic yeast fermentations [35]. We observed that co-expression of xylose metabolising enzymes alleviates this problem by facilitating the production of cellulases at fermentation temperatures and the subsequent hydrolysis of the cellulose during the fermentation. Thus a consolidated fermentation process, utilising both xylose and cellulose, is superior to either process individually.

While being a useful model for examining the activities of recombinant enzymes, the use of carbohydrate substrates like purified xylose and PASC does not reflect the complexity of natural biomass sources. Firstly, chemical or physical pre-treatments are necessary to release fermentable sugars from biomass, however, inhibitory molecules such as acetic acid, furfurals and phenols produced during this process can severely affect alcohol yields and cell growth [48]. Of the biomass sources examined in this study, spent grains appeared to be the most suitable for alcohol production, displaying high yields of both xylose and glucose. Alcohol production was inhibited by approximately 30% in fermentations carried out in SGL when compared to fermentations carried out with a purified sugar mixture containing similar ratios of xylose and glucose. While the glucose present in SGL is readily fermentable, we show that xylose utilisation is severely inhibited. The decrease in xylose utilisation was not due to a direct effect on the enzyme activities but instead appears to result from an inhibition of cell growth. Metabolome analysis of xylose utilisation in the presence of inhibitors revealed the accumulation of various non-oxidative PPP intermediates [49], indicating a bottleneck in PPP metabolism. This inhibition can be partially relieved by overexpression of a PPP transaldolase, *TAL1* [49,50]. The transcriptome analysis of cells grown in the presence of inhibitors also revealed reduced levels of certain transcripts coding for proteins not only required for carbohydrate metabolism but also, for transcriptional and translational control indicating a pleotrophic effect of inhibitors on cell metabolism [48,51].

To improve the fermentation efficiency of biomass, it is clear that the presence of inhibitors must be addressed. Chemical removal of inhibitors has been attempted [52-54], although this approach increases processing costs. Alternatively, adaptive evolutionary or directed mutagenesis approaches have been used to improve tolerance of yeast to these inhibitors [30,55-58]. The high degree of genome plasticity in *S. pastorianus* facilitates such adaptive evolutionary approaches [59,60]. Other strategies involve metabolism of the inhibitors, thus reducing the concentrations in the medium [61].

The hydrolysis of cellulose remains the rate-limiting step in bioethanol production from biomass. This coupled with the observed slower cell growth rate of yeast cultures in biomass liquor and reduced xylose utilisation impedes the development of a consolidated biomass fermentation process. Success for this goal is heavily dependent to the development of yeast strains with improved cell growth and xylose utilisation in biomass fermentations and improved cellulase production in these strains.

## Methods and materials

### Strains and media

Strain CMBS-51 is a stress tolerant derivative of the *S. pastorianus* yeast strain CMBS-33 (James et al. [59]). The tetraploid laboratory *S. cerevisiae* yeast strain (L6440) was a gift from the Fink laboratory (Massachusetts Institute of Technology, Boston, MA). The haploid *S. cerevisiae* strain BY4741 was purchased from EuroScARF. *Saccharomyces eubayanus* was obtained from the Portuguese Yeast Culture Collection (PYCC; Faculdade de Ciências e Tecnologia, Universidade Nova de Lisboa (FCT/UNL, Caparica). Yeast cultures were grown at 30°C for 24 h in Yeast Extract Peptone (YEP; 10 g litre^−1^ yeast extract, 20 g litre^−1^ peptone) supplemented with 20–50 g litre^−1^ glucose (D), sucrose (S) or xylose (X)). For plate growth assays, cultures were grown overnight in YEPD then spotted onto synthetic complete (SC) agar (20 g litre^−1^ agar, 1.7 g litre^−1^ yeast nitrogen base, 0.5 g litre^−1^ ammonium sulphate, 0.59 g litre^−1^ yeast synthetic drop-out medium, 0.2 g litre^−1^ adenine, 0.03 g litre^−1^ uracil, 0.12 g litre^−1^ lysine, 0.08 g litre^−1^ tryptophan, 0.08 g litre^−1^ leucine and 0.02 g litre^−1^ histidine) containing X or D (20 g litre^−1^) and incubated for 5 days at 30°C. For cultivation of yeast cultures bearing plasmids, the media was supplemented with hygromycin (300 μg mL^−1^) or the aminoglycoside Geneticin (G418; 200 μg mL^−1^) where appropriate. Xylose and glucose concentrations in culture media were determined using Xylose Mutarotase (Megazyme International, Bray, Ireland) or Glucose (GAHK20; Sigma-Aldrich) assay kits respectively as per manufacturers instructions. Phosphoric acid swollen cellulose (PASC) was prepared as described previously [35].

### Gene cloning and expression strategy

#### Sources of DNA

The *T. reesei* cellulase genes, *bgl1*, *egl2* and *cbh2* were previously cloned and expressed under the control of the *S. cerevisiae* Phosphoglycerate kinase (PGK) or translation elongation factor 1α (TEF) promoters and downstream CYC terminator sequence into the high copy number plasmid pRHS42H [35] (Table 1). Gene information for *xyl1* and *xdh1*, encoding for XR and XDH respectively, were sourced from The Joint Genome Institute (http://genome.jgi-psf.org/Trire2/Trire2.home.html). The genes were PCR amplified from *T. reesei* genomic DNA (QM9123) in overlapping DNA fragments using primers designed to remove intronic sequences. The *XKS1* gene sequence was amplified from *S. cerevisiae* (S288c) genomic DNA. The protein sequence for *Piromyces sp* Xylose Isomerase (xi) (www.ebi.ac.uk; ID TR:Q9P8C9_PIRSE) was reverse translated (yeast codon optimised) using GeneDesign [62]. The DNA sequence was synthesised and cloned into pUC57 by GenScript. Inc. (Piscataway, NJ).

### Modular cloning strategy

A modular gene cloning strategy as previously outlined in [63] and modified in [35] was implemented to generate single, double or triple gene expression cassettes on the high copy number plasmids pRS42H or pRSH42K, which contain genes encoding for Hygromycin or G418 aminoglycoside resistance respectively [64]. The starting plasmid was either pRS42H PGK*-bgl1-*CYCT or TEF-*bgl1-*CYCT in which the *T. reesei bgl1* gene is under the control of the *S. cerevisiae* PGK or TEF promoters respectively and upstream of the *S. cerevisiae* CYC terminator [35]. The *bgl1* gene and/or promoter were excised out by Sal1 cleavage (Table 1) and the PCR generated replacement gene cassettes inserted into the plasmid by *in vivo* homologous recombination*,* following transformation into *S. pastorianus* as described previously [35].

For multiple gene expression, gene cassette 2 (promoter, gene and terminator) was inserted at the Psi1 site on the plasmid at position −562 relative to the PGK promoter of gene cassette 1 and in the same orientation by homologous recombination *in vivo*. For triple gene expression, gene cassette 3 (promoter, gene and terminator) was inserted at the Acc65I site at position −5 relative to gene cassette 1 and in the same orientation (Table 1). The gene cassettes were amplified in one or two fragments, in the latter case with oligonucleotide primers containing overlapping sequence homology. The names of the primers used for each gene amplification are listed in Table 1 and the sequences of the oligonucleotide primers are listed in Additional file 1: Table S1.

### XDH and XR activity

Yeast strains expressing the *xyl1* and *xdh1* genes were cultured in YEPD for 24 h at 30°C. The cells were harvested, washed twice in lysis buffer (250 mM potassium phosphate pH7.0) and re-suspended in 1/10 of the original volume of the same buffer. The cells were lysed using Zirconia beads (Biospec Inc., Bartlesville, OK) with agitation at 4°C (Vibrax; IKA-Werke GmbH, Staufen, Germany) and the cell debris was removed by centrifugation. Protein concentrations of cell-free extracts were quantified using Bradford reagent and bovine serum albumin (BSA) as a standard (0 - 2 mg/mL).

XDH activity was quantified by measuring the reduction of nicotinamide adenine dinucleotide (NAD^+^) to NADH. Briefly, the cell-free extract was mixed in a 1:10 ratio with a reaction buffer consisting of 0.15 M xylitol, 0.4 mM NAD^+^ and 25 mM potassium phosphate pH 7.0. NADH production was monitored by the change in absorbance at 340 nm. XDH activity was defined as NADH (μM) produced per minute using a standard curve (0-800 μM NADH). The crude specific activity was defined as the rate of reaction divided by the protein concentration of the cell lysate (U^XDH^, μM min^−1^ mg^−1^).

The XR activity was quantified by measuring the oxidation of the reduced form of nicotinamide adenine dinucleotide phosphate (NADPH). Cell-free extract was mixed in a 1:10 ratio with a reaction buffer containing 0.34 mM NADPH, 50 mM xylose and 25 mM potassium phosphate pH 7.0 and incubated at 30°C. NADP^+^ production was monitored by the change in absorbance at 340 nm. XR activity was defined as NADPH (μM) oxidised per minute using a standard curve (0-680 μM NADPH). The crude specific activity was defined as the rate of reaction divided by the protein concentration of the cell lysate (U^XR^, μM min^−1^ mg^−1^).

### GFP quantification

Strains expressing the *xdh*-GFP gene (Table 1) were cultured in YEPD at 30°C for 24Hrs. Approximately 7x10^8^ cells were harvested by centrifugation and washed thoroughly and resuspended in 10 mL dH_2_O. The relative fluorescence (RF) of the cell sample (100 μL) was measured in a Take 3 micro-volume plate using a Synergy H1 Hybrid reader (Biotek Inc., Winooski, VT). The excitation and emission wavelengths were set at 480 nm and 510 nm respectively.

### Biomass pre-treatment

*Miscanthus sinensis* and *Miscanthus giganteus* were obtained from Dr. Trevor Hodgekinson (Botany Department, Trinity College Dublin). *Eucalyptus nitens* was supplied by Dr. Denis Lobby, DL Biotechnology, Co. Carlow. Spent grains were obtained from St James Gate Brewery, Diageo, Dublin or from the Five Lamps Microbrewery, Dublin. All biomass was dried at 60°C for 48 hrs. The stems and leaves of the plant material were separated, and then milled using a kitchen blender (Phillips) for 5 minutes at full speed. Sulphuric acid (4% w/v) was added to the dried biomass at a concentration of 5–12.5 g per 50 mL. The slurry was heated in a pressure cooker (Moulinex, Minut’ cook) on the low pressure setting for 40 minutes and then centrifuged to separate the soluble and insoluble material. The soluble fraction (liquor) was neutralised by addition of NaOH until a pH 5.5 was reached and then was passed through a Whatman No.1 filter, to remove any insoluble biomass.

### Fermentation conditions

Fermentations were carried out in sealed conical flasks at 30°C under microaerophilic conditions in medium composed of YEP (30 g L^−1^) supplemented with 412 g L^−1^ PASC, 42-50 g L^−1^ xylose, or with both 412 g L^−1^ PASC and 42-50 g L^−1^ xylose. The concentration of available glucose in 412 g L^−1^ PASC is 10.3 g L^−1^ [35]. For spent grain liquor fermentations, the neutralised liquor was supplemented with YEP (30 g L^−1^) and then filter sterilised by passage through a 0.2 μm Polyethersulfone (PES) syringe filter. The media were inoculated at a high cell density (1X10^8^ cell mL^−1^), with yeast cells pre-grown in YEPD for 24 h. Where applicable, 300 μg/mL hygromycin (Foremedium, Hunstanton, UK) or 200 μg/mL Geneticin (G418;Invivogen, San Diageo, CA, U.S.A.) was added to the cultures.

### Quantification of alcohol

Alcohol production was determined using the alcohol dehydrogenase (ADH) assay as previously described [35].

## Additional file

Additional file 1: Table S1. Sequences of Primers used for Gene Constructs.
