# Supplementary material for: Engineering Saccharomyces pastorianus for the co-utilisation of xylose and cellulose from biomass
Source: Microb Cell Fact. 2015 Apr 28;14:61. doi: 10.1186/s12934-015-0242-4 (PMC4417197; doi:10.1186/s12934-015-0242-4)
Supplement: Additional file 1: Table S1. — Sequences of Primers used for Gene Constructs. [file 12934_2015_242_MOESM1_ESM.docx]

**Sequences of Primers used for Gene Constructs**

| Plasmid | Primer | Sequence 5’>3’ |
| --- | --- | --- |
| PGK*xdh1* | xdhF  xdhR | gaattcgatatcaagcttatcgataccgtcgacaATGGCGACTCAAACGATCAACAAGG  gcgtgacataactaattacatgactcgaggtcgacTTACACCTTCTCGTTGGGCCCGGCA |
|  |  |  |
| PGK*xyl1* | xyl1F  xyl1R | gaattcgatatcaagcttatcgataccgtcgacaATGGCGTCTCCCACGCTCAAGCTCA  gcgtgacataactaattacatgactcgaggtcgacTTAGCCAAACAGGTAGAGCTTGTTG |
| PGK*xdh1*-GFP | xdhF  xdh1_GFPR  GFPF  GFPR | gaattcgatatcaagcttatcgataccgtcgacaATGGCGACTCAAACGATCAACAAGG  ACCAGTGAATAATTCTTCACCTTTAGACATCACCTTCTCGTTGGGCCCGGCAATC  ATTCTGATTGCCGGGCCCAACGAGAAGGTGATGTCTAAAGGTGAAGAATTATTCA  ACATAACTAATTACATGACTCGAGGTCGACTTATTTGTACAATTCATCCATACCA |
| PGK*xi* | xiF  xi_MidR  xi_MidF  xiR | gaattcgatatcaagcttatcgataccgtcgacaATGGCTAAGGAATACTTCCCACAAA  TCGAACCAAAGCCAATGGAACCAACCAAGCACCAA  TTGGTGCTTGGTTGGTTCCATTGGCTTTGGTTCGA  gcgtgacataactaattacatgactcgaggtcgacTTATTGGTACATAGCAACGATAGCT |
| TEF*XKS* | Psi_TEFF  xks_MidR  xks_MidF  Psi_CycR | ccaataggccgaaatcggcaaaatcccttaAATGTTTCTACTCCTTTTTTACTCT  CATATAGTGGCTAAATTATCCCCAGTCATG  CATGACTGGGGATAATTTAGCCACTATATG  cactcaaccctatctcggtctattcttttgatttaGGCCGCAAATTAAAGCCTTCGAGCG |
| PGK*xyl1*-PGK*xdh1* | Psi_PGKF  xr_MidR  xr_MidF  Psi_CycR | tttaaccaataggccgaaatcggcaaaatcccttaCCAAGAATTACTCGTGAGTAAGGAAaGA  GGCCCTTGTCGACGAGGCGCTCCATGGCGC  GCGCCATGGAGCGCCTCGTCGACAAGGGCC  cactcaaccctatctcggtctattcttttgatttaGGCCGCAAATTAAAGCCTTCGAGCG |
| PGK*xyl1*-TEF*XKS*-PGK*xdh1* | Acc_TEFF  xks_MidR  xks_MidF  AccR | atacgactcactatagggcgaattgggtacAATGTTTCTACTCCTTTTTTACTCT  CATATAGTGGCTAAATTATCCCCAGTCATG  CATGACTGGGGATAATTTAGCCACTATATG  GCGATAGTTCCTCACTCtTTCCTTACTCACGAGTAATTCTTGGGGCCGCAAATTAAAGCCTTCGAGCG |
| PGK*xyl1*-TEF*bgl1*-PGK*xdh* | Acc_TEFF  bgl1_MidR  bgl1_MidF  AccR | atacgactcactatagggcgaattgggtacAATGTTTCTACTCCTTTTTTACTCT  GGCTTCTTGAGCGGCAGGATGTTGGC  GCCAACATCCTGCCGCTCAAGAAGCC  GCGATAGTTCCTCACTCtTTCCTTACTCACGAGTAATTCTTGGGGCCGCAAATTAAAGCCTTCGAGCG |
| PGK*xyl1*-TEF*cbh2*-PGK*xdh1* | Acc_TEFF  cbh2_MidR  cbh2_MidF  AccR | atacgactcactatagggcgaattgggtacAATGTTTCTACTCCTTTTTTACTCT  CCCGCATAGTTACCGCCATTCTTGTTGGCGGTGCG  CGCACCGCCAACAAGAATGGCGGTAACTATGCGGG  GCGATAGTTCCTCACTCtTTCCTTACTCACGAGTAATTCTTGGGGCCGCAAATTAAAGCCTTCGAGCG |
| PGK*xyl1*-PGK*egl2*-PGK*xdh1* | Acc_PGKF  egl2_MidR  egl2_MidF  AccR | atacgactcactatagggcgaattgggtacAATGTTTCTACTCCTTTTTTACTCT  GGATAAACCTTCGAGGTAACGCAAGTGCCATCTGTGGTACAGCCAAAGTCAAAAC  GCGGGTTTTGACTTTGGCTGTACCACAGATGGCACTTGCGTTACCTCGAAGGTTTATCCT  GCGATAGTTCCTCACTCtTTCCTTACTCACGAGTAATTCTTGGGGCCGCAAATTAAAGCCTTCGAGCG |

Lowercase - plasmid specific sequence, UPPERCASE – gene or promoter specific sequence UNDERLINED UPPERCASE – GFP specific sequence
